# Dopamine D_2/3_ receptor antagonism reduces activity-based anorexia

**DOI:** 10.1038/tp.2015.109

**Published:** 2015-08-04

**Authors:** S J Klenotich, E V Ho, M S McMurray, C H Server, S C Dulawa

**Affiliations:** 1 Department of Psychiatry and Behavioral Neuroscience, University of Chicago, Chicago, IL, USA; 2 Department of Psychology, University of Illinois at Chicago, Chicago, IL, USA

## Abstract

Anorexia nervosa (AN) is an eating disorder characterized by severe hypophagia and weight loss, and an intense fear of weight gain. Activity-based anorexia (ABA) refers to the weight loss, hypophagia and paradoxical hyperactivity that develops in rodents exposed to running wheels and restricted food access, and provides a model for aspects of AN. The atypical antipsychotic olanzapine was recently shown to reduce both AN symptoms and ABA. We examined which component of the complex pharmacological profile of olanzapine reduces ABA. Mice received 5-HT_2A/2C_, 5-HT_3_, dopamine D_1_-like, D_2_, D_3_ or D_2/3_ antagonist treatment, and were assessed for food intake, body weight, wheel running and survival in ABA. D_2/3_ receptor antagonists eticlopride and amisulpride reduced weight loss and hypophagia, and increased survival during ABA. Furthermore, amisulpride produced larger reductions in weight loss and hypophagia than olanzapine. Treatment with either D_3_ receptor antagonist SB277011A or D_2_ receptor antagonist L-741,626 also increased survival. All the other treatments either had no effect or worsened ABA. Overall, selective antagonism of D_2_ and/or D_3_ receptors robustly reduces ABA. Studies investigating the mechanisms by which D_2_ and/or D_3_ receptors regulate ABA, and the efficacy for D_2/3_ and/or D_3_ antagonists to treat AN, are warranted.

## Introduction

Anorexia nervosa (AN) is an eating disorder characterized by hypophagia, weight loss and an intense fear of weight gain. AN typically onsets in mid-adolescence and primarily affects females.^1^ AN affects ~0.1–1.7% of the population during their lifetime.^1, 2, 3, 4, 5^ The standard mortality ratio is 5.86,^6^ representing one of the highest mortality rates of all psychiatric illnesses.^7^ No approved pharmacological treatments currently exist for AN.

The activity-based anorexia (ABA) phenomenon models aspects of AN. In the ABA paradigm, rodents housed with running wheels and subjected to restricted food access rapidly develop hypophagia, weight loss, and paradoxical increases in wheel running. Conversely, rodents exposed to either restricted food access or running wheels maintain normal body weight.^8, 9^ Progression of ABA is characterized by hypothermia, loss of estrus, increased HPA axis activity and ultimately stomach ulceration and death.^8, 9, 10, 11, 12^ ABA exhibits predictive validity for aspects of AN. For example, adolescent rodents are more vulnerable to ABA than older rodents.^13, 14, 15, 16^ Thus, the ABA paradigm provides a useful preclinical tool for studying aspects of AN.

Currently, only a handful of small, randomized controlled trials evaluating the efficacy of olanzapine to improve AN symptomatology have been performed. Three such trials reported improved AN symptomology in patients receiving olanzapine treatment,^17, 18, 19^ whereas two trials found no effect of olanzapine.^20, 21^ Thus, the potential efficacy of olanzapine in AN requires further study, and efforts to identify other treatments are imperative. Identifying the mechanisms by which olanzapine reduces ABA^22^ could provide insight into the neurobiological processes underlying AN, and thereby identify novel treatments. Olanzapine is an antagonist with high affinity for 5-HT_2A/2B/2C_, α_1_-adrenergic, muscarinic M_1__–__4,_ and histamine H_1_ receptors (*K*i=1.9–25 nM), and moderate affinity for 5-HT_3_ and dopamine D_1__–__5_ receptors (*K*i=17.1–202 nM).^23, 24^ Antagonist properties of olanzapine at either dopamine or serotonin (5-hydroxytryptamine, 5-HT) receptors could reduce ABA, as both regulate feeding^25, 26^ and locomotion.^27, 28^ Furthermore, abnormalities in 5-HT and dopamine neurotransmitter systems have been reported in ill and recovered AN patients. For example, the major metabolite of 5-HT, 5-hydroxyindoleacetic acid, is elevated after weight restoration in AN.^29, 30^ Recovered AN patients also exhibit reductions in homovanillic acid, a dopamine metabolite,^31^ and increases in D_2/3_ receptor binding in the anteroventral striatum.^32^ These biomarkers may represent trait alterations that increase vulnerability to AN. As ill and recovered AN patients exhibit alterations in serotonergic and dopaminergic signaling systems, olanzapine may reduce ABA in rodents and AN symptoms in patients through antagonist properties at certain serotonergic and/or dopaminergic receptors.

To determine which aspect of olanzapine's complex pharmacology reduces ABA, we examined the effects of selective serotonergic or dopaminergic receptor antagonists on ABA in mice. We assessed the effects of the 5-HT_2A/2C_ receptor antagonist ritanserin, and the 5-HT_3_ receptor antagonist ondansetron. Then, we examined the effects of the D_1_-like antagonist SCH23390 and the D_2/3_ receptor antagonists eticlopride or amisulpride. We also compared the ability of amisulpride and olanzapine to reduce ABA. Finally, we used the D_3_ receptor antagonist SB277011A and the D_2_ receptor antagonist L-741,626 to discern the effects of selective D_3_ and D_2_ receptor antagonism on ABA.

## Materials and methods

### Animals

Balb/cJ female mice (Jackson Laboratories, Bar Harbor, ME, USA), aged 8 weeks, were acclimated to the vivarium for 1 week before the experimental procedures. Mice received *ad libitum* access to standard chow and water, except during food restriction. Animals were euthanized, or ‘dropped', from experiments when they lost 25% of their baseline body weight (assessed on the last day of baseline). All the procedures were conducted in accord with the National Institutes of Health laboratory animal care guidelines and with Institutional Animal Care and Use Committee approval at the University of Chicago.

### Experimental conditions

Mice were housed in a climate-controlled room maintained on a 12:12 light–dark cycle (lights off at 2000 h). Cages (19.56 × 34.70 × 14.41 cm) were equipped with wireless low-profile running wheels (Med Associates, St Albans, VT, USA). Running wheels transmitted running data every 30 s to a computer with Wheel Manager Software 24 h a day. Food was provided in a glass jar (65 cm diameter × 50 cm height) during baseline and restriction periods.

### Activity-based anorexia paradigm

The mice were pseudo-randomly divided into experimental groups on the basis of body weight upon arrival. During acclimation (2 days), baseline (7 days), and food restriction (14 days), mice were singly housed and given 24 h running wheel access. During restriction, the mice received daily food access for 6 h a day beginning at 0900 h. Six hours daily food access induces a dropout rate of approximately ⩽7 days, permitting detection of either increases or decreases in survival.^22^ Daily body weight, food intake and wheel running were recorded during baseline and restriction conditions. Food anticipatory activity (FAA), defined as running activity 0–4 h before food access, and postprandial activity (PPA), defined as running activity following food access and before initiation of the dark cycle, were also determined. Days to dropout (loss of 25% baseline body weight) provided a measure of survival.

### Experimental design

For all the experiments, the mice were subjected to the treatment regime (see Supplementary Methods) and ABA paradigm described above. Sample sizes were chosen on the basis of previous studies, which identified statistical differences in survival in the ABA paradigm.^22^ The experimenter was not blind to the treatment groups to accurately dose each group via the drinking water (See Supplementary Methods).

Experiment 1: mice received 0, 0.01, 0.1 or 1 mg kg^−1^ per day ritanserin and a separate group received 25 mg kg^−1^ per day olanzapine as a positive control (*n*=8 per group). Ritanserin^33, 34, 35^ and olanzapine^22^ doses were based on previous studies.

Experiment 2: mice received 0 (*n*=12), 0.1 (*n*=11), 1 (*n*=12) or 10 mg kg^−1^ per day ondansetron (*n*=12),^36, 37, 38^ or 30 mg kg^−1^ per day olanzapine (*n*=12).^22^

Experiment 3: mice received 0, 0.005, 0.5 or 0.5 mg kg^−1^ per day SCH23390,^39, 40, 41, 42^ or 15 mg kg^−1^ per day olanzapine^22^ (*n*=12 per group).

Experiment 4: mice received 0, 0.1, 0.5 or 1 mg kg^−1^ per day eticlopride,^43, 44, 45^ or 35 mg kg^−1^ per day olanzapine^22^ (*n*=12 per group).

Experiment 5: mice received 0, 10, 50 or 100 mg kg^−1^ per day amisulpride.^46, 47, 48, 49^ A separate group received 1 mg kg^−1^ per day eticlopride^43, 44, 45^ as a positive control (*n*=12 per group).

Experiment 6: effects of amisulpride (0, 100 or 150 mg kg^−1^ per day)^46, 47, 48, 49^ were compared with olanzapine (12 or 18 mg kg^−1^ per day)^22^ (*n*=12 per group).

Experiment 7: mice received 0 (*n*=15), 5 (*n*=14), 25 (*n*=15) or 50 mg kg^−1^ per day (*n*=15) SB277011A.^50, 51, 52, 53^ For experiments 7 and 8, the restriction period lasted only 10 days as dropout from the ABA paradigm generally occurs within this timeframe.

Experiment 8: mice received 0, 1, 10 or 20 mg kg^−1^ per day L-741,626 (*n*=15 per group).^54, 55, 56, 57^

### Statistics

For baseline data, analyses of variance assessed the effects of treatment as a between-subjects factor, and day as a within-subjects factor for each dependent variable (body weight, food intake, wheel running). *Post hoc* analyses of variance resolved main effects of treatment or interactions of treatment × day. Bonferroni adjustments were made when *post hoc* analyses of variance were applied.

The mice drop out of the ABA paradigm during restriction, creating data sets with missing values. Therefore, general linear models (PROC GLIMMIX; SAS v9.2, SAS Institute, Cary, NC, USA; code available from corresponding author upon request) were used to assess differences in body weight, food intake, wheel running, FAA, and PPA during restriction. *Post hoc* analyses resolving main effects of treatment and treatment × day interactions were adjusted for multiple comparisons using the false discovery rate method. Survival analysis was performed using the Kaplan–Meier test with Logrank (Mantel–Cox) and Peto–Peto–Wilcoxon *post hoc* tests.

For baseline and restriction data, all the *post hoc* tests compared drug doses to vehicle. For experiment 6, all the doses were compared with one another. The mice were excluded from the data set when their log-transformed dropout day was >2 s.d. from the group mean (see Supplementary Results). Significance was set at *P*<0.05.

## Results

### Ritanserin reduces food intake and wheel running during ABA

During baseline, neither ritanserin nor olanzapine treatment altered body weight (Supplementary Table S1). Main effects indicated that 25 mg kg^−1^ per day olanzapine reduced food intake (F_(4,31)_=4.098, *P*<0.01) and wheel running (F_(4,32)_=3.020, *P*<0.05; all comparisons *P*<0.05, Supplementary Table S1). For experiment 1, baseline running-wheel data reflects the last 4 days of baseline due to a malfunction of the data acquisition system.

**Figure 1:**
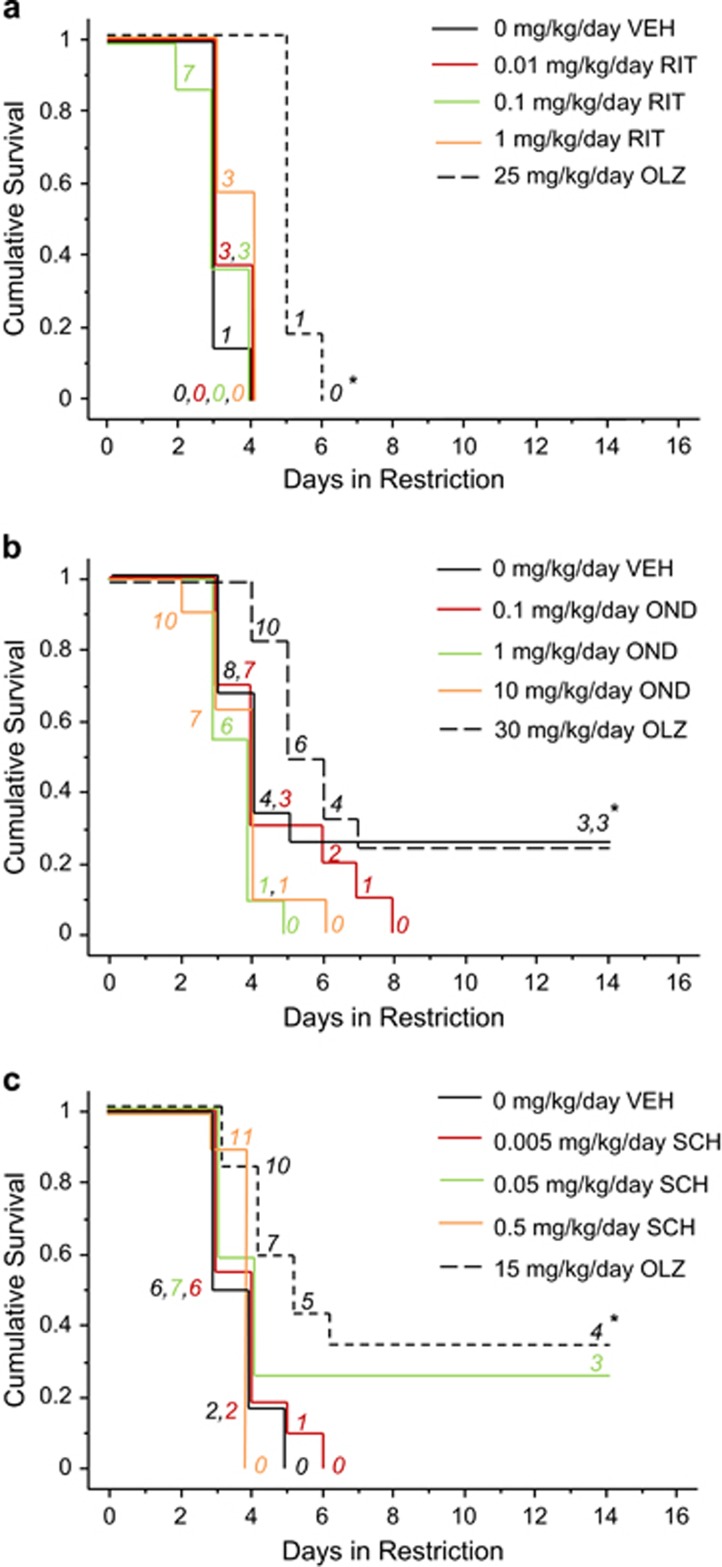
Ritanserin, ondansetron and SCH23390 treatment do not increase survival in ABA. Survival curves during restriction of (**a**) ritanserin-, (**b**) ondansetron- and (**c**) SCH23390-treated mice in comparison with vehicle- or olanzapine-treated mice. Numbers in italics represent mice remaining in food restriction. Asterisk indicates olanzapine is significantly different from vehicle (*P*<0.05). BL, baseline; OLZ, olanzapine; OND, ondansetron; RIT, ritanserin; SCH, SCH23390; VEH, vehicle.

During food restriction, ritanserin treatment did not alter survival (Figure 1a). Conversely, 25 mg kg^−1^ per day olanzapine treatment increased survival (*P*<0.0005).

A main effect of treatment (F_(4,111)_=5.23, *P*<0.001) and *post hoc* analyses revealed that 25 mg kg^−1^ per day olanzapine reduced weight loss (*P*<0.005), while ritanserin had no effect (Supplementary Figure S1A). Specifically, 25 mg kg^−1^ per day olanzapine increased body weight on days 1–3 relative to vehicle (treatment × day, F_(21,111)_=17.04, *P*<0.0001; all comparisons *P*<0.001; Supplementary Figure S1A). Olanzapine 25 mg kg^−1^ per day also protected against hypophagia (F_(4,112)_=12.50, *P*<0.0001; all comparisons *P*<0.0001; Supplementary Figure S1B). Specifically, 25 mg kg^−1^ per day olanzapine increased food intake on day 1, while 0.1 mg kg^−1^ per day and 1 mg kg^−1^ per day ritanserin reduced food intake on days 3 and 4, and day 4, respectively, relative to vehicle (treatment × day, F_(22,112)_=13.69, *P*<0.0001; all comparisons *P*<0.05; Supplementary Figure S1B).

All doses of ritanserin and 25 mg kg^−1^ per day olanzapine reduced wheel running (F_(4,112)_=4.91, *P*<0.005; all comparisons, *P*<0.05; Supplementary Figure S1C). Specifically, 0.01, 0.1 and 1 mg kg^−1^ per day ritanserin reduced wheel running on day 4, days 2–4 and day 4, respectively (treatment × day, F_(22,112)_=4.06, *P*<0.0001; all comparisons *P*<0.05; Supplementary Figure S1C). All the doses of ritanserin reduced FAA on days 3 and 4, and 25 mg kg^−1^ per day olanzapine reduced FAA on day 4 (treatment × day, F_(17,81)_=4.79, *P*<0.0001; all comparisons *P*<0.05; Supplementary Figure S1D). Finally, 0.1 mg kg^−1^ per day ritanserin reduced PPA on day 3 (treatment × day, F_(22,110)_=5.99, *P*<0.0001; all comparisons *P*<0.05; Supplementary Figure S1E).

### Ondansetron treatment decreases body weight and food intake during ABA

During baseline, neither ondansetron nor olanzapine altered body weight or food intake (Supplementary Table S1). However, a main effect of treatment (F_(4,50)_=4.275, *P*<0.005) and *post hoc* analyses indicated that 30 mg kg^−1^ per day olanzapine reduced wheel running (*P*<0.05, Supplementary Table S1).

During restriction, ondansetron did not alter survival (Figure 1b). However, 30 mg kg^−1^ per day olanzapine increased survival early in the restriction period (*P*<0.05), but in not the late phase (*P*=0.25).

All the doses of ondansetron and olanzapine produced further reductions in body weight (F_(4,244)_=13.85, *P*<0.0001; all comparisons *P*<0.0001; Supplementary Figure S2A) and food intake (F_(4,245)_=102.99, *P*<0.0001; all comparisons *P*<0.0001; Supplementary Figure S2B) compared with vehicle. Specifically, 0.1, 1 and 10 mg kg^−1^ per day ondansetron reduced food intake on days 6 and 7, days 4 and 5 and days 3–6, respectively (treatment × day, F_(46,245)_=23.43, *P*<0.0001; all comparisons *P*<0.05). Finally, 30 mg kg^−1^ per day olanzapine reduced food intake on days 5, 6 and 13 (all comparisons *P*<0.05; Supplementary Figure S2B).

Neither ondansetron nor olanzapine altered wheel running (Supplementary Figure S2C) or FAA (Supplementary Figure S2D). However, all the doses of ondansetron increased PPA (F_(4,245)_=4.53, *P*<0.005; all comparisons *P*<0.05; Supplementary Figure S2E, see inset). Specifically, 0.1 mg kg^−1^ per day ondansetron increased PPA on day 6 (treatment × day, F_(46,245)_=3.65, *P*<0.0001; all comparisons *P*<0.05; Supplementary Figure S2E).

### SCH23390 treatment increases body weight and food intake during ABA

Neither SCH23390 nor 15 mg kg^−1^ per day olanzapine altered the body weight, food intake or wheel running during baseline (Supplementary Table S1).

During restriction, SCH23390 did not alter survival (Figure 1c). Conversely, 15 mg kg^−1^ per day olanzapine increased survival (*P*<0.01).

A main effect of treatment (F_(4,245)_=36.12, *P*<0.0001) revealed that 0.05 and 0.5 mg kg^−1^ per day SCH23390 and 15 mg kg^−1^ per day olanzapine reduced weight loss (all comparisons *P*<0.001; Supplementary Figure S3A, see inset). Specifically, 0.05 mg kg^−1^ per day SCH23390 and 15 mg kg^−1^ per day olanzapine increased the body weight on day 5 and days 3–5, respectively (treatment × day, F_(42,245)_=23.77, *P*<0.0001; all comparisons *P*<0.05; Supplementary Figure S3A). Furthermore, 0.005 mg kg^−1^ per day and 0.05 mg kg^−1^ per day SCH23390, as well as 15 mg kg^−1^ per day olanzapine reduced hypophagia (F_(4,239)_=89.41, *P*<0.0001; all comparisons *P*<0.001; Supplementary Figure S3B, see inset).

Wheel running was not altered by either treatment (Supplementary Figure S3C). However, both 0.05 mg kg^−1^ per day SCH23390 and 15 mg kg^−1^ per day olanzapine increased FAA (F_(4,198)_=25.09, *P*<0.0001; all comparisons *P*<0.005; Supplementary Figure S3D, see inset). In addition, 0.05 mg kg^−1^ per day SCH23390 reduced PPA (F_(4,244)_=13.86, *P*<0.0001; all comparisons *P*<0.0005; Supplementary Figure S3E, see inset). Finally, 0.005 mg kg^−1^ per day SCH23390 and 15 mg kg^−1^ per day olanzapine increased PPA on day 2, and days 1 and 2, respectively (treatment × day, F_(42,244)_=5.97, *P*<0.0001; all comparisons *P*<0.05; Supplementary Figure S3E).

### Eticlopride treatment robustly increases survival in ABA

During baseline, neither eticlopride nor olanzapine altered body weight, food intake or wheel running (Supplementary Table S1).

**Figure 2:**
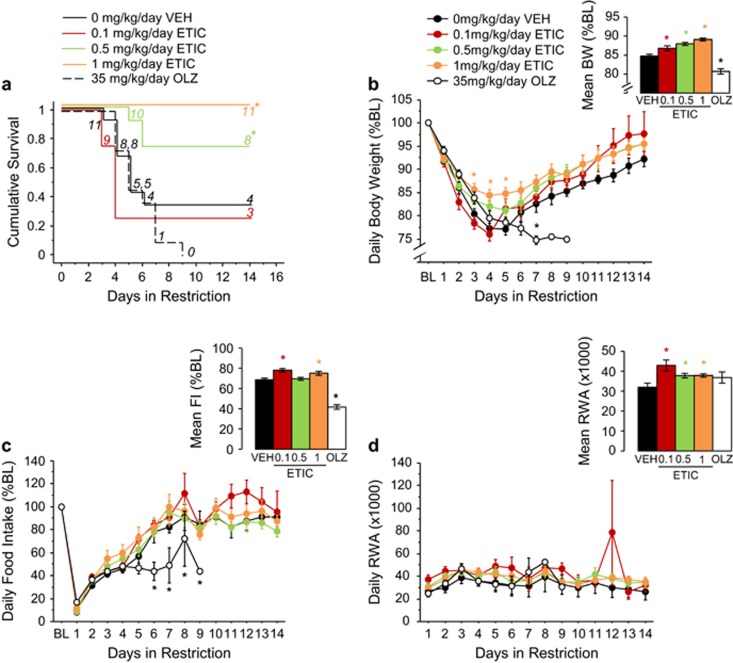
Eticlopride treatment robustly reduces ABA behavior. (**a**) Survival curve, (**b**) body weight, (**c**) food intake and (**d**) running-wheel activity during restriction in vehicle-, eticlopride- or olanzapine-treated mice. Results are expressed as mean±s.e.m. Insets indicate mean±s.e.m. during restriction for the dependent measure depicted. Numbers in italics represent mice remaining in food restriction. Asterisk color indicates which group is significantly different from vehicle (*P*<0.05). Black asterisk refers to olanzapine. BL, baseline; BW, body weight; ETIC, eticlopride; FI, food intake; OLZ, olanzapine; RWA, running-wheel activity; VEH, vehicle.

During restriction, 0.5 and 1 mg kg^−1^ per day eticlopride increased survival (all comparisons *P*<0.05; Figure 2a). Importantly, 1 mg kg^−1^ per day eticlopride completely prevented dropout. Mice receiving 0.1 mg kg^−1^ per day eticlopride or 35 mg kg^−1^ per day olanzapine did not differ from mice receiving vehicle.

All the doses of eticlopride protected against weight loss (F_(4,448)_=36.31, *P*<0.0001; all comparisons *P*<0.01; Figure 2b, see inset). Conversely, 35 mg kg^−1^ per day olanzapine reduced body weight (*P*<0.0001). In addition, 1 mg kg^−1^ per day eticlopride increased body weight on days 3–5, while 35 mg kg^−1^ per day olanzapine reduced body weight on day 7 (treatment × day, F_(64,448)_=14.91, *P*<0.0001; all comparisons *P*<0.05; Figure 2b). Similarly, 0.1 and 1 mg kg^−1^ per day eticlopride increased, whereas 35 mg kg^−1^ per day olanzapine decreased food intake (F_(4,446)_=47.54, *P*<0.0001; all comparisons *P*<0.05; Figure 2c, see inset). Specifically, 35 mg kg^−1^ per day olanzapine reduced food intake on days 6–9 (treatment × day, F_(64,446)_=26.81, *P*<0.0001; all comparisons *P*<0.05; Figure 2c).

All the doses of eticlopride increased wheel running (F_(4,449)_=2.88, *P*<0.05; all comparisons *P*<0.05; Figure 2d, see inset). No effects of treatment on FAA were found (Supplementary Figure S4A). However, 35 mg kg^−1^ per day olanzapine increased PPA (F_(4,449)_=8.55, *P*<0.0001; all comparisons *P*<0.001; Supplementary Figure S4B, see inset).

### Amisulpride treatment increases survival, body weight and food intake during ABA

During baseline, neither amisulpride nor eticlopride altered the body weight or food intake (Supplementary Table S1). However, 1 mg kg^−1^ per day eticlopride reduced wheel running on day 1 (treatment × day, F_(24,53)_=2.685, *P*<0.0001; data not shown).

**Figure 3:**
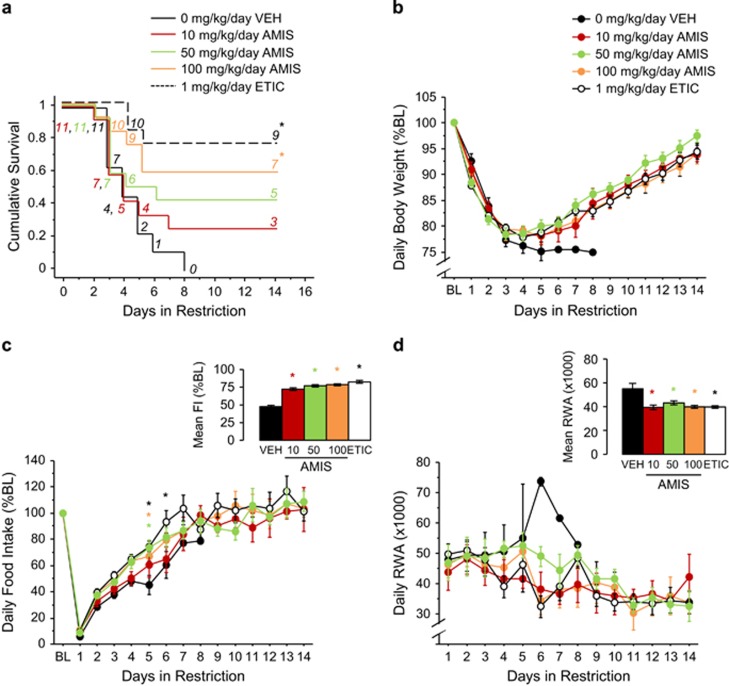
Amisulpride treatment robustly reduces ABA behavior. (**a**) Survival curve, (**b**) body weight, (**c**) food intake and (**d**) running-wheel activity during restriction in vehicle-, amisulpride- and eticlopride-treated mice. Results are expressed as mean±s.e.m. Insets indicate mean±s.e.m. during restriction for the dependent measure depicted. Numbers in italics represent mice remaining in food restriction. Asterisk color indicates which group is significantly different from vehicle (*P*<0.05). Black asterisk refers to eticlopride. AMIS, amisulpride; BL, baseline; BW, body weight; ETIC, eticlopride; FI, food intake; OLZ, olanzapine; RWA, running-wheel activity; VEH, vehicle.

During restriction, amisulpride increased survival (Figure 3a). Although 10 and 50 mg kg^−1^ per day amisulpride induced nonsignificant increases in survival, 100 mg kg^−1^ per day amisulpride increased survival (*P*<0.05). Similarly, 1 mg kg^−1^ per day eticlopride increased survival (*P*<0.0005).

All the treatments reduced weight loss (F_(4,408)_=12.10, *P*<0.0001; all comparisons *P*<0.0001; Figure 3b) and hypophagia (F_(4,406)_=51.29, *P*<0.0001; all comparisons *P*<0.0001; Figure 3c, see inset). Furthermore, 50 and 100 mg kg^−1^ per day amisulpride increased food intake on day 5, and 1 mg kg^−1^ per day eticlopride increased food intake on food restriction days 5 and 6 (treatment × day, F_(63,406)_=35.92, *P*<0.0001; all comparisons *P*<0.05; Figure 3c).

All the treatments reduced wheel running (F_(4,394)_=3.45, *P*<0.01; all comparisons *P*<0.05; Figure 3d, see inset). Moreover, both 100 mg kg^−1^ per day amisulpride and 1 mg kg^−1^ per day eticlopride reduced FAA on food restriction day 3, and food restriction days 3 and 5, respectively (treatment × day, F_(62,359)_=3.54, *P*<0.0001; all comparisons *P*<0.05; Supplementary Figure S5A). Neither amisulpride nor eticlopride altered PPA (Supplementary Figure S5B).

### Amisulpride produces larger reductions in body weight loss and hypophagia during ABA than olanzapine

Neither amisulpride nor olanzapine altered body weight, food intake or wheel running during baseline (Supplementary Table S1).

**Figure 4:**
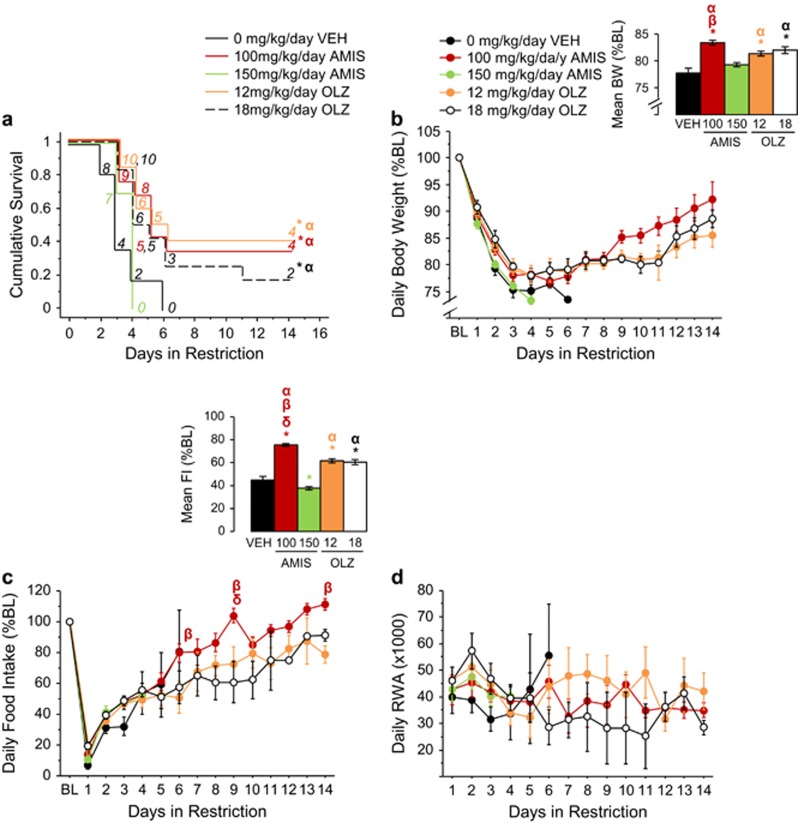
Comparison of amisulpride and olanzapine treatment on ABA behavior. (**a**) Survival curve, (**b**) body weight, (**c**) food intake and (**d**) running-wheel activity during restriction in vehicle-, amisulpride- and olanzapine-treated mice. Results are expressed as mean±s.e.m. Insets indicate mean±s.e.m. during restriction for the dependent measure depicted. Numbers in italics represent mice remaining in food restriction. Symbol color indicates which group is significantly different from vehicle (*P<0.05), 150 mg kg^−1^ per day amisulpride (α *P*<0.05), 12 mg kg^−1^ per day olanzapine (β *P*<0.05) and 18 mg kg^−1^ per day olanzapine (δ *P*<0.05). Black symbols refer to 18 mg kg^−1^ per day olanzapine. AMIS, amisulpride; BL, baseline; BW, body weight; FI, food intake; OLZ, olanzapine; RWA, running-wheel activity; VEH, vehicle.

During restriction, both amisulpride and olanzapine increased survival compared with vehicle (Figure 4a). Specifically, 100 mg kg^−1^ per day (*P*<0.05), but not 150 mg kg^−1^ per day, amisulpride increased survival. The 12 mg kg^−1^ per day (*P*<0.01) and 18 mg kg^−1^ per day (*P*<0.05) doses of olanzapine also increased survival. The 100 mg kg^−1^ per day amisulpride group did not differ from the 12 mg kg^−1^ per day or 18 mg kg^−1^ per day olanzapine groups. However, both the olanzapine treatment groups increased survival compared with 150 mg kg^−1^ per day amisulpride (all comparisons *P*<0.05). Finally, 100 mg kg^−1^ per day amisulpride increased survival compared with 150 mg kg^−1^ per day amisulpride (*P*<0.05), while 12 and 18 mg kg^−1^ per day olanzapine produced comparable survival times.

The 100 mg kg^−1^ per day dose of amisulpride and 12 and 18 mg kg^−1^ per day doses of olanzapine reduced weight loss compared with vehicle during restriction (F_(4,281)_=15.60, *P*<0.0001; all comparisons *P*<0.001; Figure 4b, see inset). Moreover, 100 mg kg^−1^ per day amisulpride reduced weight loss relative to 12 mg kg^−1^ per day olanzapine and 150 mg kg^−1^ per day amisulpride (all comparisons *P*<0.005). Both the olanzapine doses reduced weight loss relative to 150 mg kg^−1^ per day amisulpride (all comparisons *P*<0.005). Furthermore, 100 mg kg^−1^ per day amisulpride reduced hypophagia compared with all the other treatment groups (F_(4,280)_=107.40, *P*<0.0001; all comparisons *P*<0.0001; Figure 4c, see inset). Both the doses of olanzapine reduced hypophagia relative to 150 mg kg^−1^ per day amisulpride and vehicle (all comparisons *P*<0.0005). On the other hand, 150 mg kg^−1^ per day amisulpride reduced food intake compared with vehicle (*P*<0.05). Furthermore, 100 mg kg^−1^ per day amisulpride increased food intake compared with 12 mg kg^−1^ per day olanzapine on days 6, 9 and 14, and 18 mg kg^−1^ per day olanzapine on restriction day 9 (treatment × day, F_(51,280)_=28.10, *P*<0.0001; all comparisons *P*<0.05; Figure 4c).

Neither amisulpride nor olanzapine altered wheel running (Figure 4d). A main effect of treatment (F_(4,233)_=11.68, *P*<0.0001) showed that 100 mg kg^−1^ per day amisulpride increased FAA compared with 150 mg kg^−1^ per day amisulpride, 18 mg kg^−1^ per day olanzapine and vehicle (all comparisons *P*<0.05; Supplementary Figure S6A, see inset). Also, 12 mg kg^−1^ per day olanzapine increased FAA relative to 18 mg kg^−1^ per day olanzapine, 150 mg kg^−1^ per day amisulpride and vehicle (all comparisons *P*<0.05). Finally, 18 mg kg^−1^ per day olanzapine increased FAA compared with 150 mg kg^−1^ per day amisulpride and vehicle (all comparisons *P*<0.05). Neither amisulpride nor olanzapine altered PPA (Supplementary Figure S6B).

### SB277011A treatment increases survival in ABA

During baseline, SB277011A did not alter body weight, food intake or wheel running (Supplementary Table S1).

**Figure 5:**
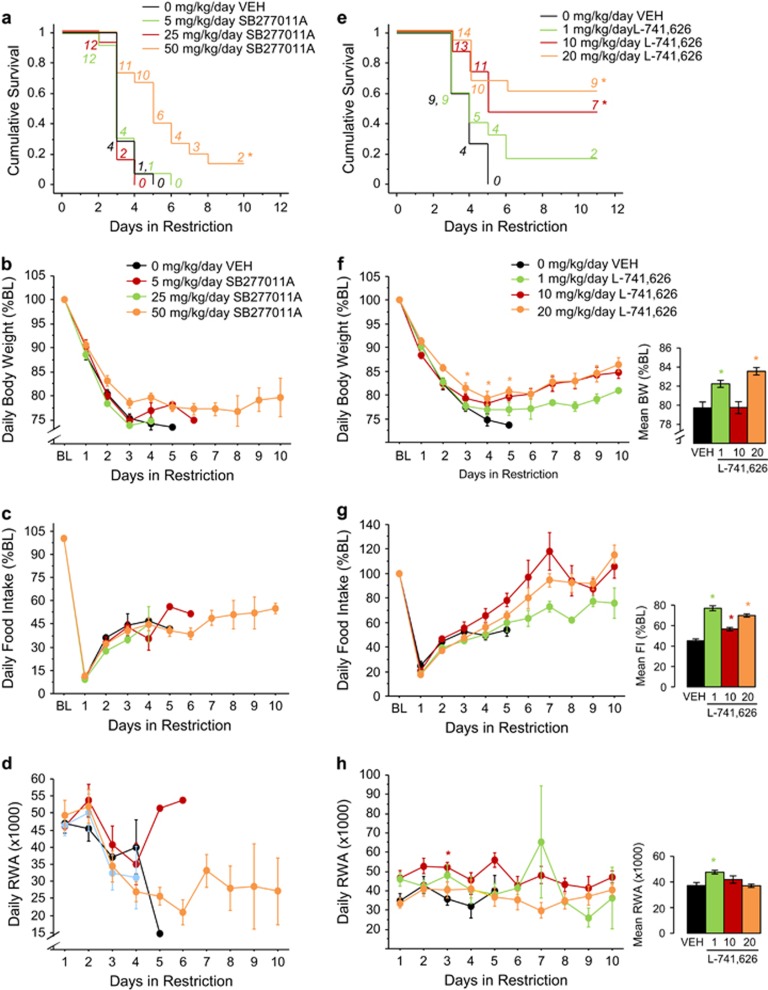
SB277011A and L-741,626 treatment reduce ABA behavior. (**a**) Survival curve, (**b**) body weight, (**c**) food intake (**d**) and running-wheel activity of vehicle- or SB277011A-treated mice. (**e**) Survival curve, (**f**) body weight, (**g**) food intake (**h**) and running-wheel activity of vehicle- or L-741,626-treated mice. Results are expressed as mean±s.e.m. Insets indicate mean±s.e.m. during restriction for the dependent measure depicted. Numbers in italics represent mice remaining in food restriction. Asterisk color indicates which group is significantly different from vehicle (*P*<0.05). BL, baseline; BW, body weight; FI, food intake; RWA, running-wheel activity; VEH, vehicle.

During restriction, 50 mg kg^−1^ per day SB277011A increased survival (*P*<0.005; Figure 5a). Neither body weight (Figure 5b) nor food intake (Figure 5c) were altered by SB277011A.

Furthermore, SB277011A did not induce changes in wheel running (Figure 5d) or FAA (Supplementary Figure S7A). Conversely, a main effect of treatment (F_(3,193)_=6.43, *P*<0.0005) indicated that both 25 and 50 mg kg^−1^ per day SB277011A increased PPA (all comparisons *P*<0.05; Supplementary Figure S7B, see inset).

### L-741,626 treatment increases survival, body weight and food intake during ABA

During baseline, L-741,626 did not alter body weight, food intake or wheel running (Supplementary Table S1).

During restriction, both 10 mg kg^−1^ per day (*P*<0.05) and 20 mg kg^−1^ per day (*P*<0.0005) L-741,626 increased survival (Figure 5e).

Both 1 mg kg^−1^ per day and 20 mg kg^−1^ per day L-741,626 reduced weight loss (F_(3,305)_=14.55, *P*<0.0001; all comparisons *P*<0.001; Figure 5f, see inset). Specifically, 20 mg kg^−1^ per day L-741,626 increased body weight on days 3–5 (treatment × day, F_(34,305)_=13.28, *P*<0.0001; all comparisons *P*<0.05; Figure 5f). Furthermore, all the doses of L-714,626 reduced hypophagia (F_(3,301)_=56.25, *P*<0.0001; all comparisons *P*<0.0001; Figure 5g, see inset).

A main effect of treatment (F_(3,305)_=11.40; *P*<0.0001) showed that 1 mg kg^−1^ per day L-741,626 increased wheel running (*P*<0.001; Figure 5h, see inset). Furthermore, 10 mg kg^−1^ per day L-741,626 increased wheel running on day 3 (treatment × day, F_(34,305)_=2.15, *P*<0.001; *P*<0.05; Figure 5h). Both 1 mg kg^−1^ per day and 10 mg kg^−1^ per day L-741,626 increased FAA (F_(3,264)_=4.80, *P*<0.005; all comparisons *P*<0.05; Supplementary Figure S7C, see inset). Moreover, all the doses of L-741,626 decreased PPA (F_(3,305)_=12.95, *P*<0.0001; all comparisons *P*<0.05; Supplementary Figure S7D, see inset). Specifically, 1 mg kg^−1^ per day L-741,626 decreased PPA on day 5, whereas 10 mg kg^−1^ per day L-741,626 increased PPA on day 1 (treatment × day, F_(34,305)_=5.69, *P*<0.0001; all comparisons *P*<0.05). Finally, 10 and 20 mg kg^−1^ per day L-741,626 decreased PPA on days 4 and 5, and day 5, respectively (all comparisons *P*<0.0005; Supplementary Figure S7D).

## Discussion

Here we report that selective antagonism of D_2_ and/or D_3_ receptors significantly reduces ABA. The D_2/3_ receptor antagonists eticlopride and amisulpride robustly increased survival, and reduced weight loss and hypophagia during restriction. Thus, D_2/3_ receptor blockade underlies, at least in part, the ability of olanzapine to reduce ABA. Furthermore, amisulpride produced larger reductions in weight loss and hypophagia than olanzapine, suggesting that dopamine D2/3 receptor antagonism may underlie the mechanism of action of olanzapine. Thus, D2/3 antagonists should be examined in AN. Furthermore, selective antagonism of either D_2_ or D_3_ receptors also reduced ABA. Since selective D_3_ antagonism has a reduced propensity to induce extrapyramidal symptoms (EPS),^53, 55, 58, 59, 60^ selective D_3_ receptor antagonism should also be explored as a novel treatment for AN. Although D_1_-like antagonist SCH23390 increased body weight and food intake during restriction, survival was not increased. Surprisingly, 5-HT_2A/2C_ or 5-HT_3_ receptor antagonism increased ABA. In sum, D_2/3_ and/or D_3_ receptor antagonists may provide effective treatment for AN, and should be investigated in clinical trials.

Increases in survival are paramount to indicating reductions in ABA. Additional effects indicating reduced ABA include reductions in weight loss and hypophagia. Reductions in wheel running during restriction can also indicate reduced ABA, but animals also run less before dropout, confounding the interpretation of wheel running. Recently, Wu *et al.*^61^ has shown that decreases in FAA and increases in PPA correlate with weight loss^61^ contrary to previous findings, which indicated that decreases in FAA are associated with reduced ABA.^22, 62, 63^ Our findings indicate that changes in FAA and PPA remain inconsistent or unobserved, and do not always correlate with increases in survival. For example, amisulpride decreased FAA in experiment 5 (Supplementary Figure S5A), but increased FAA in experiment 6 (Supplementary Figure S6A), despite consistently increasing survival (Figures 3a and 4a). Moreover, none of our experiments yielded simultaneous increases in FAA and decreases in PPA alongside increases in survival.

Overall, none of the selective serotonergic or dopaminergic antagonists used herein produced significant alterations in the body weight, food intake or wheel running during baseline. Therefore, observed effects on ABA were unlikely owing to drug-induced changes in metabolism, activity levels or appetite, but were specific to restriction conditions.

We used a range of olanzapine doses across the present studies to identify an optimal dose of olanzapine. Doses from 12 to 25 mg kg^−1^ per day increased survival (Figures 1a, c and 4a), whereas higher doses did not (Figures 1b and 2a). Furthermore, optimal doses of olanzapine (12–18 mg kg^−1^ per day) not only increased survival (Figures 1c and 4a), but also increased body weight (Supplementary Figures S3A and Figure 4b) and food intake (Supplementary Figures S3B and Figure 4b), during food restriction. Moreover, as olanzapine doses above 25 mg kg^−1^ per day increased, ABA also increased. For example, both 30 and 35 mg kg^−1^ per day olanzapine decreased body weight and food intake during restriction (Supplementary Figures S2A and S2B and Figures 2b and c). Furthermore, decreases in baseline food intake (25 mg kg^−1^ per day) and wheel running (30 mg kg^−1^ per day) indicate a propensity for higher doses of olanzapine to induce nonspecific effects (Supplementary Table S1).

Olanzapine has a higher affinity for 5-HT_2A/2C_ receptors than dopamine receptors,^23, 24^ and 5-HT_2A/2C_ receptor blockade is thought to contribute to weight gain following atypical antipsychotic treatment.^64^ However, the effects of 5-HT_2A/2C_ antagonists on food intake in rodents vary, and are dependent on experimental conditions.^65, 66, 67, 68, 69^ Our findings show that ritanserin had no effect on survival, and reduced food intake (0.1 and 1 mg kg^−1^ per day) and wheel running (all doses)(Figure 1a, Supplementary Figures S1A and S1C). Thus, 5-HT_2A/2C_ receptor antagonism may increase ABA by increasing hypophagia during restriction.

Ondansetron reduces symptoms of bulimia nervosa^70^ and obsessive-compulsive disorder,^71^ two disorders comorbid with AN.^72^ However, ondansetron did not increase survival in ABA and exacerbated ABA by reducing body weight and food intake (Supplementary Figures S2A and 2B). Therefore, 5-HT_3_ receptor antagonism is unlikely to contribute to the ability of olanzapine to reduce ABA.

Olanzapine has high affinity binding at 5-HT_6_ receptors (*K*i=6 nM).^24^ However, mice have reduced 5-HT_6_ receptor expression in the striatum compared with rats and humans, and alterations in key amino-acid sequences, which result in different pharmacological profiles.^73^ Thus, findings obtained using mice might not be readily translated to humans. Therefore, we did not investigate the effects of 5-HT_6_ antagonists on ABA.

Although antagonism of D_1_-like receptors using SCH23390 did not alter survival, 0.05 mg kg^−1^ per day and 0.5 mg kg^−1^ per day SCH23390 reduced weight loss and 0.005 mg kg^−1^ per day and 0.05 mg kg^−1^ per day SCH23390 reduced hypophagia (Figure 1c, Supplementary Figures S3A and S3B). As SCH23390 induced only minor reductions in ABA without increasing survival, D_1_-like receptor antagonism is unlikely to contribute substantially to the ability of olanzapine to reduce ABA.

The D_2/3_ receptor antagonists eticlopride and amisulpride robustly increased survival in ABA. Notably, 1 mg kg^−1^ per day eticlopride prevented mice from dropping out of restriction (Figure 2a). Moreover, both amisulpride and eticlopride reduced weight loss and hypophagia (Figures 3a–c). Although optimal doses of amisulpride and olanzapine required to reduce ABA produced comparable increases in survival, 100 mg kg^−1^ per day amisulpride produced larger reductions in weight loss and hypophagia than olanzapine (Figures 4a–c). These findings suggest that dopamine D2/3 receptor antagonism plays a significant role in the therapeutic mechanism of olanzapine to reduce ABA behavior.

As D_2/3_ antagonists robustly reduced ABA, we examined whether blockade of one or both dopamine receptors drives this effect. We found that selective antagonism of either D_2_ or D_3_ receptors increased survival (Figures 5a and e). The D_2_ receptor antagonist L-741,626 also reduced weight loss and hypophagia during restriction (Figures 5f and g). Increases in survival induced by D_3_ antagonist SB277011A appear smaller in magnitude, and might be due to nonsignificant reductions in weight loss and wheel running (Figures 5b and d). Higher doses of SB277011A might produce more robust increases in survival. Although L-741,626 and SB277011A exhibit ~40-fold and 100-fold selectivity for the D_2_ or D_3_ receptor, respectively, cross-receptor reactivity may have been present at the doses used in these studies. Recent findings in rats indicate that L-741,626 has relatively no D_3_ receptor occupancy at 10 mg kg^−1^ per day, and that 10 mg kg^−1^ per day SB277011A exhibits ~20% receptor occupancy at D_2_ receptors.^74^ Given that survival increased as the dose of SB277011A increased, D_2_ receptor antagonism might have a role in the ability of SB277011A to increase survival. Future studies using conditional knockout of D2 or D3 receptors will be required to definitively resolve their respective contributions to ABA behavior.

Clinical trials evaluating the efficacy of dopaminergic antagonists to ameliorate AN symptomology are limited and severely underpowered. Overall, typical antipsychotic treatment produces marginal increases in body weight in AN patients, as well as EPS and sedation.^75, 76^ Conversely, atypical antipsychotics, most accurately distinguished from typical antipsychotics by a reduced susceptibility to EPS,^77^ show promise in reducing AN symptomology. Consistent with our finding that D_2/3_ antagonists reduce ABA, a small, single-blind trial showed that atypical antipsychotic amisulpride increased weight gain in AN patients.^78^ Conversely, the related compound sulpiride, produced only marginal increases in weight gain in anorexic patients.^79^ These data are consistent with the report that recovered AN patients show increased binding affinity at D_2/3_ receptors in the anteroventral striatum, which could be a trait alteration that increases susceptibility to AN.^32^ The preferential high affinity binding at limbic cortical D_2/3_ receptors, rather than striatal D_2/3_ receptors, may underlie the reduced incidence of EPS following amisulpride treatment.^49, 80, 81^ Furthermore, amisulpride does not show high affinity binding at 5-HT_2C_ receptors, muscarinic, or histamine H_1_ receptors,^81, 82^ which are thought to contribute to the metabolic side effects of olanzapine.^83^ Therefore, amisulpride may reduce AN symptomology with fewer side effects associated with olanzapine or typical antipsychotic treatment.

We also found that selective antagonism of D_3_ receptors reduces ABA (Figure 5a). Since antagonism at striatal D_2_ receptors is thought to drive EPS,^77^ the enriched mesolimbic expression of D_3_ receptors^84^ may indicate a novel treatment target for AN with reduced susceptibility to EPS. In fact, reduced EPS were observed in humans,^58^ and in the catalepsy rodent model of EPS,^53^ following D_3_ antagonist treatment. As both amisulpride and D_3_ antagonists are thought to produce a low incidence of EPS, these agents may provide therapeutic benefit in AN while avoiding unwanted side effects.

We aimed to determine which aspect of olanzapine's complex pharmacology reduces ABA by examining the effects of selective serotonergic or dopaminergic receptor antagonists on ABA. Our findings suggest that D_2/3_ receptor antagonism mediates reductions in ABA produced by olanzapine, while D_1_-like, 5-HT_2A/2C,_ and 5-HT_3_ receptor blockade does not (Supplementary Table S3). However, complex interactions between multiple receptors could have a role in the mechanism of olanzapine to reduce ABA. We did not examine the effects of simultaneously antagonizing three or more receptors on ABA, yet we still identified robust effects of D_2/3_ receptor blockade via two selective D_2/3_ receptor antagonists, and selective antagonism at D_2_ or D_3_ receptors alone.

In conclusion, we show that D_2/3_ antagonists robustly increase survival, body weight, and food intake during ABA and therefore might provide effective treatment for AN. Furthermore, we found that D_3_ receptor antagonism alone also reduces ABA, and could provide a novel approach to treating AN with reduced risk for EPS. Currently, there is a dearth of suitably powered, placebo-controlled clinical trials evaluating potential pharmacological treatments for anorexia nervosa. Our data indicate that clinical trials evaluating the efficacy of amisulpride and D_3_ antagonists in AN are warranted.
