# Supplementary material for: Dopamine D2/3 receptor antagonism reduces activity-based anorexia
Source: Transl Psychiatry. 2015 Aug 4;5(8):e613–. doi: 10.1038/tp.2015.109 (PMC4564564; doi:10.1038/tp.2015.109)
Supplement: Supplementary Information [file tp2015109x1.doc]

**SUPPLEMENTAL INFORMATION**

**Supplemental Methods**

**Chemicals**

All drugs were administered in the drinking water in dark bottles throughout the baseline (7 days) and food restriction periods (21 days). Olanzapine (Sequoia Research Products, Pangbourne, United Kingdom) was dissolved in a minimal quantity of glacial acetic acid (0.12%), adjusted to a pH of 5.7-5.8 with sodium hydroxide, and raised to volume with distilled water. The same proportion of glacial acetic acid was added to vehicle control, ritanserin (Sigma Aldrich, St. Louis, MO, USA), and ondansetron (Sigma Aldrich, St. Louis, MO, USA) solutions, which were also adjusted to a pH of 5.7-5.8. Eticlopride (Sigma Aldrich, St. Louis, MO, USA), SCH23390 (Tocris Bioscience, Bristol, UK), and SB277011A (Tocris Bioscience, Bristol, UK) were dissolved in distilled water. Amisulpride (Sequoia Research Products, Pangbourne, United Kingdom) was dissolved in distilled water with a minimal quantity of hydrochloric acid (0.04%) and gentle warming, adjusted to pH 7 with sodium hydroxide, and raised to volume with distilled water. L-741,626 (Tocris Bioscience, Bristol, UK) was dissolved in distilled water with a minimal quantity of lactic acid (0.15%), adjusted to pH 6.0-6.1 with sodium hydroxide, and raised to volume with distilled water. The same proportion of lactic acid was added to the vehicle control solution. Drug concentrations were adjusted from stock solutions to account for changes in water intake and body weight twice weekly during the baseline period, and daily during the food restriction period. Ritanserin, SCH 23390, and SB277011A stocks were changed daily. Olanzapine, ondansetron, eticlopride, amisulpride, and L-741,626 stocks were changed biweekly. Average dose of drug treatment and water intake during baseline and food restriction are presented in Table S2.

**Supplemental Results**

In each experiment, mice were excluded from the dataset when their drop out day (loss of 25% baseline body weight during the restriction period) fell 2 standard deviations from the mean of the log of the drop day within treatment group. Outliers in survival from each experiment are as follows: in experiment 1, one mouse from the vehicle, 25mg/kg/day olanzapine, and 1mg/kg/day ritanserin treated groups was removed from data analyses. During experiment 2, one mouse from the 0.1mg/kg/day, 1mg/kg/day, and 10mg/kg/day ondansetron treated groups was removed from data analyses. Two mice from the 0.5mg/kg/day and one mouse from the 0.005mg/kg/day SCH23390 groups were removed from all analyses in experiment 3. Three mice, one in the 0.5mg/kg/day eticlopride group, one in the 1mg/kg/day eticlopride group, and one in the 35mg/kg/day olanzapine group, were removed from all data analyses in experiment 4. One mouse from the vehicle group was removed from data analyses in experiment 5. One mouse in the vehicle group and two in the 150mg/kg/day amisulpride group were removed from all analyses in experiment 6. In experiment 7, four mice, one from the vehicle and 5mg/kg/day SB277011A groups, and two from the 25mg/kg/day SB277011A group, were removed from all analyses. Finally, in experiment 8, two mice from the 1mg/kg/day L-741,626 group were removed from all analyses. The overall results from each experiment are presented in Table S3.

**Supplemental Table and Figure Legends**

**Table S1. Baseline dependent measures for all experiments.** Body weight, food intake, and running wheel activity during baseline in mice treated with ritanserin, ondansetron, SCH 23390, eticlopride, amisulpride, amisulpride or olanzapine, SB 277011A, and L-741,626. Values are means ± SEM. *(p<0.05) compared to vehicle treatment. BW, body weight; FI, food intake; RWA, running wheel activity; VEH, vehicle; RIT, ritanserin; OLZ, olanzapine; OND, ondansetron; SCH, SCH 23390; ETIC, eticlopride; AMIS, amisulpride.

**Table S2. Average drug dosage and intake during baseline and restriction.** Average drug treatment dose and water intake during baseline and food restriction for mice treated with ritanserin, ondansetron, SCH 23390, eticlopride, amisulpride, SB277011A, L-741,626, and olanzapine during each experiment. Values are means ± SEM. Dose is presented as mg/kg/day and water intake is presented in milliliters. RIT, ritanserin; OLZ, olanzapine; OND, ondansetron; SCH, SCH 23390; ETIC, eticlopride; AMIS, amisulpride.

**Table S3. Summary of restriction dependent measures for each experiment.** Arrows and symbols of inequality depict the direction of significant change for each dependent measure during restriction of each experiment. ND, no difference; RIT, ritanserin; OLZ, olanzapine; OND, ondansetron; SCH, SCH 23390; ETIC, eticlopride; AMIS, amisulpride.

**Figure S1. Effects of ritanserin and olanzapine treatment on ABA behavior. (A)** Body weight, **(B)** food intake, **(C)** running wheel activity, **(D)** FAA, and **(E)** PPA during restriction in ritanserin- and olanzapine-treated mice. Results expressed as mean ± SEM. *Significant difference from vehicle (p<0.05). Asterisk color indicates which group is significantly different from vehicle (p<0.05). Black asterisk refers to olanzapine. BL, baseline; VEH, vehicle; RIT, ritanserin; OLZ, olanzapine; RWA, running wheel activity; FAA, food anticipatory activity; PPA, postprandial activity.

**Figure S2. Effects of ondansetron and olanzapine treatment on ABA behavior. (A)** Body weight, **(B)** food intake, **(C)** running wheel activity, **(D)** FAA, and **(E)** PPA during restriction in ondansetron- and olanzapine-treated mice. Results expressed as mean ± SEM. Inset indicates mean ± SEM during restriction for the dependent measure depicted. *Significant difference from vehicle (p<0.05). Asterisk color indicates which group is significantly different from vehicle (p<0.05). Black asterisk refers to olanzapine. BL, baseline; VEH, vehicle; OND, ondansetron; OLZ, olanzapine; RWA, running wheel activity; FAA, food anticipatory activity; PPA, postprandial activity.

**Figure S3. Effects of SCH 23390 and olanzapine treatment on ABA behavior. (A)** Body weight, **(B)** food intake, **(C)** running wheel activity, **(D)** FAA, and **(E)** PPA during restriction in SCH223390- and olanzapine- treated mice. Results expressed as mean ± SEM. Insets indicate mean ± SEM during restriction for the dependent measure depicted. *Significant difference from vehicle (p<0.05). Asterisk color indicates which group is significantly different from vehicle (p<0.05). Black asterisk refers to olanzapine. BL, baseline; VEH, vehicle; SCH, SCH 23390; OLZ, olanzapine; RWA, running wheel activity; FAA, food anticipatory activity; PPA, postprandial activity.

**Figure S4. Effects of eticlopride and olanzapine treatment on FAA and PPA. (A)** FAA and **(B)** PPA during restriction in eticlopride- and olanzapine-treated mice. Results expressed as mean ± SEM. Inset indicates mean ± SEM during restriction for the dependent measure depicted. *Significant difference from vehicle (p<0.05). VEH, vehicle; ETIC, eticlopride; OLZ, olanzapine; FAA, food anticipatory activity; PPA, postprandial activity.

**Figure S5. Effects of amisulpride and eticlopride treatment on FAA and PPA. (A)** FAA and **(B)** PPA during restriction in amisulpride- and eticlopride-treated mice. Results expressed as mean ± SEM. *Significant difference from vehicle (p<0.05). Asterisk color indicates which group is significantly different from vehicle (p<0.05). Black asterisk refers to eticlopride. VEH, vehicle; AMIS, amisulpride; ETIC, eticlopride; FAA, food anticipatory activity; PPA, postprandial activity.

**Figure S6. Effects of amisulpride and olanzapine treatment on FAA and PPA. (A)** FAA and **(B)** PPA during restriction in amisulpride- and olanzapine-treated mice. Results expressed as mean ± SEM. Inset indicates mean ± SEM during restriction for the dependent measure depicted. *Significant difference from vehicle (p<0.05). ^α^Significant difference from 150 mg/kg/day amisulpride (p<0.05). ^β^Significant difference from 12 mg/kg/day olanzapine (p<0.05). ^δ^Significant difference from 18 mg/kg/day olanzapine (p<0.05). Symbol color indicates which group is significantly different from vehicle (*), 150 mg/kg/day amisulpride (α), 12 mg/kg/day olanzapine (β), and 18 mg/kg/day olanzapine (δ) (p<0.05). Black symbols refer to 18mg/kg/day olanzapine. VEH, vehicle; AMIS, amisulpride; OLZ, olanzapine; FAA, food anticipatory activity; PPA, postprandial activity.

**Figure S7. Effects of SB 277011A and L-741,626 treatment on FAA and PPA. (A)** FAA and **(B)** PPA during restriction in SB 277011A-treated mice (n=60, 14-15 per group). **(C)** FAA and **(D)** PPA during restriction in L-741,626-treated mice. Results expressed as mean ± SEM. Insets indicate mean ± SEM during restriction for the dependent measure depicted. *Significant difference from vehicle (p<0.05). Asterisk color indicates which group is significantly different from vehicle (p<0.05). VEH, vehicle; FAA, food anticipatory activity; PPA, postprandial activity.
