# Supplementary material for: Dopamine D2/3 receptor antagonism reduces activity-based anorexia
Source: Transl Psychiatry. 2015 Aug 4;5(8):e613–. doi: 10.1038/tp.2015.109 (PMC4564564; doi:10.1038/tp.2015.109)
Supplement: Supplementary Table 1 [file tp2015109x2.doc]

| **Baseline Dependent Measures for All Experiments** | | | |
| --- | --- | --- | --- |
| Experiment 1 | BW | FI | RWA |
| 0 mg/kg/day VEH | 18.55 ± 0.19 | 3.89 ± 0.09 | 33256.78 ± 2384.65 |
| 0.01 mg/kg/day RIT | 18.22 ± 0.14 | 3.73 ± 0.10 | 33052.53 ± 2497.56 |
| 0.1 mg/kg/day RIT | 18.04 ± 0.09 | 3.73 ± 0.08 | 23522.50 ± 2193.42 |
| 1 mg/kg/day RIT | 18.01 ± 0.11 | 3.65 ± 0.10 | 33533.54 ± 2280.82 |
| 25 mg/kg/day OLZ | 17.58 ± 0.12 | 3.17 ± 0.07 * | 18670.68 ± 1435.43 * |
| Experiment 2 | BW | FI | RWA |
| 0 mg/kg/day VEH | 19.25 ± 0.11 | 4.02 ± 0.08 | 34299.76 ± 1361.46 |
| 0.1 mg/kg/day OND | 19.21 ± 0.14 | 3.83 ± 0.10 | 31072.39 ± 1390.09 |
| 1 mg/kg/day OND | 19.40 ± 0.12 | 4.02 ±0.09 | 32982.49 ± 1390.24 |
| 10 mg/kg/day OND | 19.32 ± 0.10 | 4.15 ± 0.09 | 39490.36 ± 1186.60 |
| 30 mg/kg/day OLZ | 18.91 ± 0.12 | 3.76 ± 0.07 | 26199.92 ± 957.44 * |
| Experiment 3 | BW | FI | RWA |
| 0 mg/kg/day VEH | 19.23 ± 0.13 | 4.11 ± 0.11 | 37945.35 ± 1419.23 |
| 0.005 mg/kg/day SCH | 19.01 ± 0.15 | 4.06 ± 0.11 | 37947.65 ± 1260.00 |
| 0.05 mg/kg/day SCH | 19.32 ± 0.14 | 3.77 ± 0.10 | 35802.51 ± 1616.46 |
| 0.5 mg/kg/day SCH | 19.11 ± 0.12 | 3.96 ± 0.11 | 37187.49 ± 1586.62 |
| 15 mg/kg/day OLZ | 18.71 ± 0.13 | 3.55 ± 0.09 | 28755.19 ± 1304.38 |
| Experiment 4 | BW | FI | RWA |
| 0 mg/kg/day VEH | 18.40 ± 0.11 | 3.65 ± 0.08 | 30174.05 ± 1569.99 |
| 0.1 mg/kg/day ETIC | 18.89 ± 0.09 | 3.77 ± 0.07 | 36659.81 ± 1156.61 |
| 0.5 mg/kg/day ETIC | 18.84 ± 0.17 | 3.83 ± 0.08 | 34435.69 ± 1044.87 |
| 1 mg/kg/day ETIC | 18.72 ± 0.11 | 3.80 ± 0.10 | 29514.38 ± 1181.76 |
| 35 mg/kg/day OLZ | 18.15 ± 0.18 | 3.49 ± 0.09 | 23461.44 ± 1028.84 |
| Experiment 5 | BW | FI | RWA |
| 0 mg/kg/day VEH | 19.82 ± 0.14 | 4.14 ± 0.08 | 39382.35 ± 1003.69 |
| 10 mg/kg/day AMIS | 20.00 ± 0.12 | 3.80 ± 0.10 | 35311.39 ± 1273.17 |
| 50 mg/kg/day AMIS | 19.90 ± 0.12 | 3.66 ± 0.11 | 29396.43 ± 1718.72 |
| 100 mg/kg/day AMIS | 20.02 ± 0.12 | 3.71 ± 0.10 | 33700.38 ± 1202.01 |
| 1 mg/kg/day ETIC | 19.543 ± 0.14 | 3.66 ± 0.09 | 33824.42 ± 1227.55 |
| Experiment 6 | BW | FI | RWA |
| 0 mg/kg/day VEH | 18.86 ± 0.14 | 3.56 ± 0.08 | 34609.25 ± 1705.41 |
| 100 mg/kg/day AMIS | 19.17 ± 0.16 | 3.42 ± 0.09 | 26269.41 ± 1637.31 |
| 150 mg/kg/day AMIS | 19.82 ± 0.15 | 3.66 ± 0.09 | 28543.26 ± 1379.32 |
| 12 mg/kg/day OLZ | 18.67 ± 0.13 | 3.62 ± 0.12 | 30074.00 ± 1261.57 |
| 18 mg/kg/day OLZ | 18.65 ± 0.11 | 3.79 ± 0.09 | 32317.56 ± 1040.21 |
| Experiment 7 | BW | FI | RWA |
| 0 mg/kg/day VEH | 19.11 ± 0.11 | 3.85 ± 0.09 | 41959.54 ± 1077.45 |
| 5 mg/kg/day SB277011A | 19.14 ± 0.09 | 3.98 ± 0.09 | 43331.95 ± 964.34 |
| 25 mg/kg/day SB277011A | 19.00 ± 0.08 | 4.01 ± 0.08 | 41490.79 ± 1194.41 |
| 50 mg/kg/day SB277011A | 18.61 ± 0.09 | 3.61 ± 0.07 | 38863.76 ± 1115.15 |
| Experiment 8 | BW | FI | RWA |
| 0 mg/kg/day VEH | 18.80 ± 0.12 | 3.44 ± 0.08 | 29408.66 ± 1424.53 |
| 1 mg/kg/day L-741,626 | 18.89 ± 0.12 | 3.66 ± 0.07 | 35766.54 ± 1060.70 |
| 10 mg/kg/day L-741,626 | 19.22 ± 0.10 | 3.35 ± 0.05 | 27279.39 ± 913.34 |
| 20 mg/kg/day L-741,626 | 19.00 ± 0.11 | 3.36 ± 0.07 | 22935.57 ± 770.09 |
