# Supplementary material for: Dopamine D2/3 receptor antagonism reduces activity-based anorexia
Source: Transl Psychiatry. 2015 Aug 4;5(8):e613–. doi: 10.1038/tp.2015.109 (PMC4564564; doi:10.1038/tp.2015.109)
Supplement: Supplementary Table 2 [file tp2015109x3.doc]

| **Group** | **Dose (mg/kg/day)** | | **Water Intake (mL)** | |
| --- | --- | --- | --- | --- |
| **Experiment 1** | **Baseline** | **Restriction** | **Baseline** | **Restriction** |
| RIT 0.01 mg/kg/day | 0.0124 ± 0.0002 | 0.0096 ± 0.0016 | 4.2925 ± 0.5000 | 4.2218 ± 0.6945 |
| RIT 0.1 mg/kg/day | 0.1248 ± 0.0017 | 0.1185 ± 0.0231 | 4.4863 ± 0.4688 | 4.5404 ± 0.5984 |
| RIT 1mg/kg/day | 1.2712 ± 0.1564 | 0.9636 ± 0.0748 | 4.5014 ± 0.2357 | 3.9336 ± 0.3342 |
| OLZ 25 mg/kg/day | 29.2232 ± 19.1239 | 28.4936 ± 2.0374 | 1.8849 ± 0.5680 | 3.2736 ± 0.2121 |
| **Experiment 2** | **Baseline** | **Restriction** | **Baseline** | **Restriction** |
| OND 0.1 mg/kg/day | 0.1086 ± 0.0066 | 0.1074 ± 0.0112 | 4.6662 ± 0.0613 | 5.8737 ± 0.4281 |
| OND 1 mg/kg/day | 1.1485 ± 0.0349 | 0.9778 ± 0.1981 | 4.8080 ± 0.2666 | 5.2931 ± 0.9264 |
| OND 10 mg/kg/day | 11.2544 ± 0.8675 | 10.6701 ± 1.2621 | 4.9442 ± 0.1303 | 5.0674 ± 1.0660 |
| OLZ 30 mg/kg/day | 27.3366 ± 3.3312 | 32.0983 ± 2.5316 | 3.2778 ± 0.1027 | 3.9961 ± 0.2417 |
| **Experiment 3** | **Baseline** | **Restriction** | **Baseline** | **Restriction** |
| SCH 0.005 mg/kg/day | 0.0055 ± 0.0009 | 0.0054 ± 0.0004 | 4.6654 ± 0.1863 | 5.0725 ± 0.2387 |
| SCH 0.05 mg/kg/day | 0.0535 ± 0.0067 | 0.0518 ± 0.0027 | 4.2212 ± 0.1627 | 5.8952 ± 0.1941 |
| SCH 0.5 mg/kg/day | 0.5275 ± 0.0060 | 0.5597 ± 0.0708 | 4.1923 ± 0.1012 | 4.9133 ± 0.3358 |
| OLZ 15 mg/kg/day | 14.2079 ± 2.0630 | 15.9177 ± 1.2357 | 2.9099 ± 0.1430 | 4.5898 ± 0.2979 |
| **Experiment 4** | **Baseline** | **Restriction** | **Baseline** | **Restriction** |
| ETIC 0.1 mg/kg/day | 0.1188 ± 0.0126 | 0.1001 ± 0.0048 | 3.9847 ± 0.0992 | 4.5703 ± 0.1492 |
| ETIC 0.5 mg/kg/day | 0.5120 ± 0.0150 | 0.5110 ± 0.0150 | 3.5852 ± 0.0275 | 4.8094 ± 0.0793 |
| ETIC 1 mg/kg/day | 1.0328 ± 0.0325 | 1.0288 ± 0.0286 | 3.3584 ± 0.0246 | 1.2504 ± 0.0878 |
| OLZ 35 mg/kg/day | 30.9038 ± 5.3997 | 38.7623 ± 3.6043 | 2.3514 ± 0.0386 | 3.6507 ± 0.2072 |
| **Experiment 5** | **Baseline** | **Restriction** | **Baseline** | **Restriction** |
| AMIS 10 mg/kg/day | 10.6438 ± 0.0967 | 10.4163 ± 0.4479 | 3.8918 ± 0.1399 | 5.9877 ± 0.1105 |
| AMIS 50 mg/kg/day | 53.0824 ± 0.8083 | 51.9359 ± 2.3943 | 2.8485 ± 0.2044 | 5.7680 ± 0.1056 |
| AMIS 100 mg/kg/day | 113.2308 ± 0.3407 | 103.6978 ± 3.7577 | 3.8892 ± 0.3034 | 5.7441 ± 0.1032 |
| ETIC 1 mg/kg/day | 1.0111 ± 0.0360 | 1.0245 ± 0.0310 | 3.6589 ± 0.1044 | 4.7579 ± 0.1149 |
| **Experiment 6** | **Baseline** | **Restriction** | **Baseline** | **Restriction** |
| AMIS 100 mg/kg/day | 90.7469 ± 0.5066 | 108.7578 ± 7.4024 | 3.8689 ± 0.1089 | 5.8355 ± 0.1675 |
| AMIS 150 mg/kg/day | 134.3788 ± 5.5455 | 193.5666 ± 32.3671 | 4.1707 ± 0.2164 | 5.8168 ± 0.1430 |
| OLZ 12 mg/kg/day | 10.5796 ± 0.7271 | 12.9432 ± 0.8273 | 3.5061 ± 0.2920 | 4.6723 ± 0.1729 |
| OLZ 18 mg/kg/day | 15.9399 ± 2.4022 | 19.1453 ± 1.4264 | 3.5231 ± 0.1782 | 3.7444 ± 0.2118 |
| **Experiment 7** | **Baseline** | **Restriction** | **Baseline** | **Restriction** |
| SB277011A 5 mg/kg/day | 4.7136 ± 0.2110 | 5.9070 ± 0.6351 | 3.8141 ± 0.0188 | 5.6806 ± 0.4086 |
| SB277011A 25 mg/kg/day | 25.2921 ± 0.7710 | 28.5106 ± 1.5880 | 4.0838 ± 0.0928 | 4.5972 ± 0.0746 |
| SB277011A 50 mg/kg/day | 49.8518 ± 3.3189 | 49.3036 ± 3.6901 | 3.4002 ± 0.0669 | 3.1248 ± 0.2560 |
| **Experiment 8** | **Baseline** | **Restriction** | **Baseline** | **Restriction** |
| L-741,626 1 mg/kg/day | 0.9904 ± 0.0098 | 1.0407 ± 0.0636 | 3.9752 ± 0.0320 | 5.0560 ± 0.1464 |
| L-741,626 10 mg/kg/day | 9.1486 ± 0.2208 | 10.5040 ± 0.3206 | 3.5112 ± 0.1615 | 3.9695 ± 0.0959 |
| L-741,626 20 mg/kg/day | 17.7618 ± 0.5397 | 21.1186 ± 0.6087 | 3.0771 ± 0.0961 | 3.3913 ± 0.1547 |
