# Supplementary material for: Dopamine D2/3 receptor antagonism reduces activity-based anorexia
Source: Transl Psychiatry. 2015 Aug 4;5(8):e613–. doi: 10.1038/tp.2015.109 (PMC4564564; doi:10.1038/tp.2015.109)
Supplement: Supplementary Table 3 [file tp2015109x4.doc]

| Experiment | Treatment | Survival | BW | FI | RWA | FAA | PPA |
| --- | --- | --- | --- | --- | --- | --- | --- |
| 1 | RIT vs VEH | ND | ND | ↓ | ↓ | ↓ | ↓ |
|  | 25 mg/kg/day OLZ vs VEH | ↑ | ↑ | ↑ | ND | ND | ND |
| 2 | OND vs VEH | ND | ↓ | ↓ | ND | ND | ↑ |
|  | 30 mg/kg/day OLZ vs VEH | ↑ | ↓ | ↓ | ND | ND | ND |
| 3 | SCH vs VEH | ND | ↑ | ↑ | ND | ↑ | ↑↓ |
|  | 15 mg/kg/day OLZ vs VEH | ↑ | ↑ | ↑ | ND | ↑ | ↑ |
| 4 | ETIC vs VEH | ↑ | ↑ | ↑ | ↑ | ND | ND |
|  | 35 mg/kg/day OLZ | ND | ↓ | ↓ | ND | ND | ↑ |
| 5 | AMIS vs VEH | ↑ | ↑ | ↑ | ↓ | ↓ | ND |
|  | 1 mg/kg/day ETIC vs VEH | ↑ | ↑ | ↑ | ↓ | ↓ | ND |
| 6 | AMIS vs VEH | ↑ | ↑ | ↑ | ND | ↑ | ND |
|  | OLZ vs VEH | ↑ | ↑ | ↑ | ND | ↑ | ND |
|  | AMIS vs OLZ | ND | AMIS>OLZ | AMIS>OLZ | ND | AMIS>OLZ | ND |
| 7 | EXP 7: SB277011A vs VEH | ↑ | ND | ND | ND | ND | ↑ |
| 8 | EXP 8: L-741,626 vs VEH | ↑ | ↑ | ↑ | ↑ | ↑ | ↓ |
