# Supplementary figures and images for: Dopamine D2/3 receptor antagonism reduces activity-based anorexia
Source: Transl Psychiatry. 2015 Aug 4;5(8):e613–. doi: 10.1038/tp.2015.109 (PMC4564564; doi:10.1038/tp.2015.109)

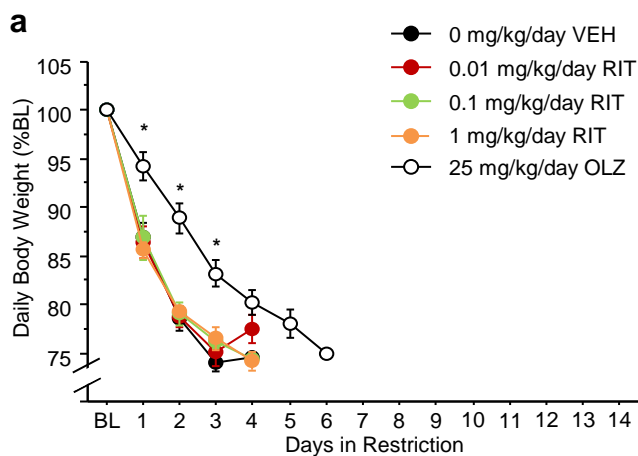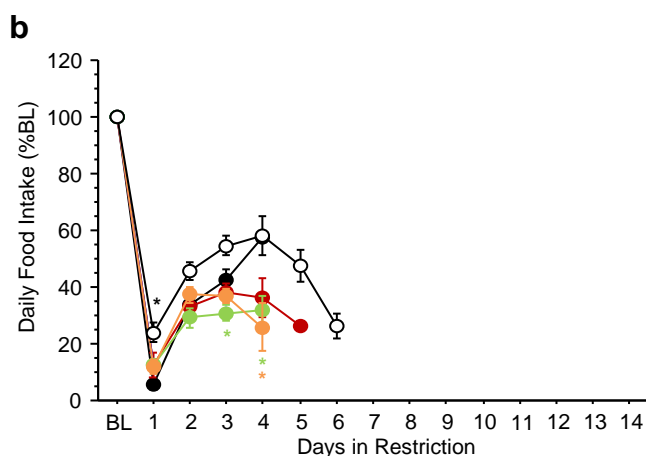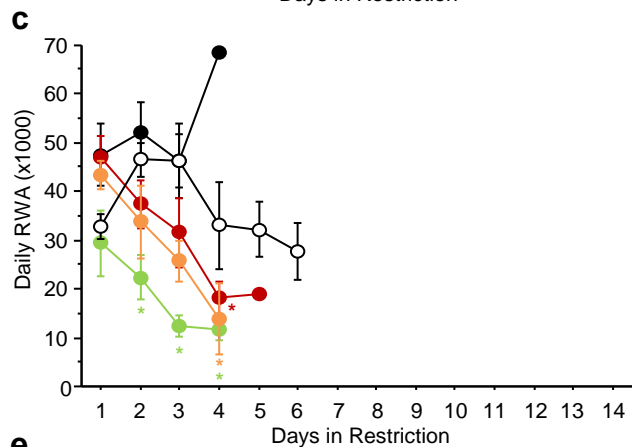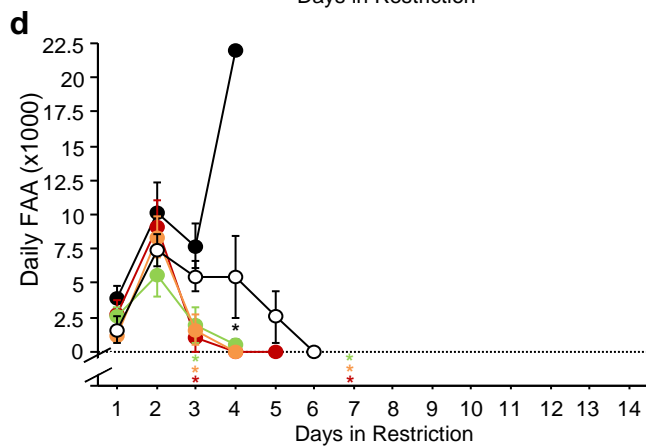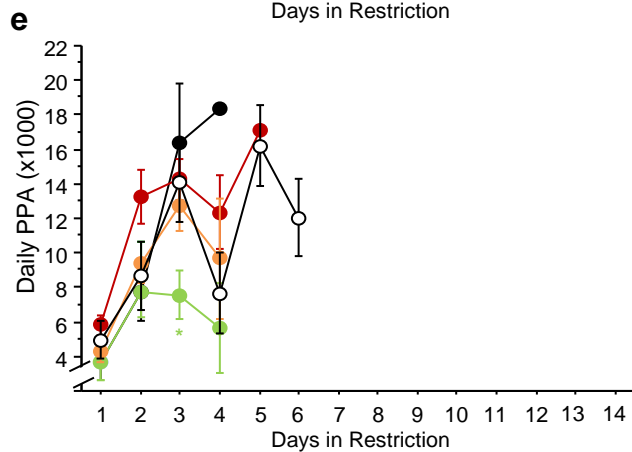

Supplement: Supplementary Figure 1 [file tp2015109x5.pdf]

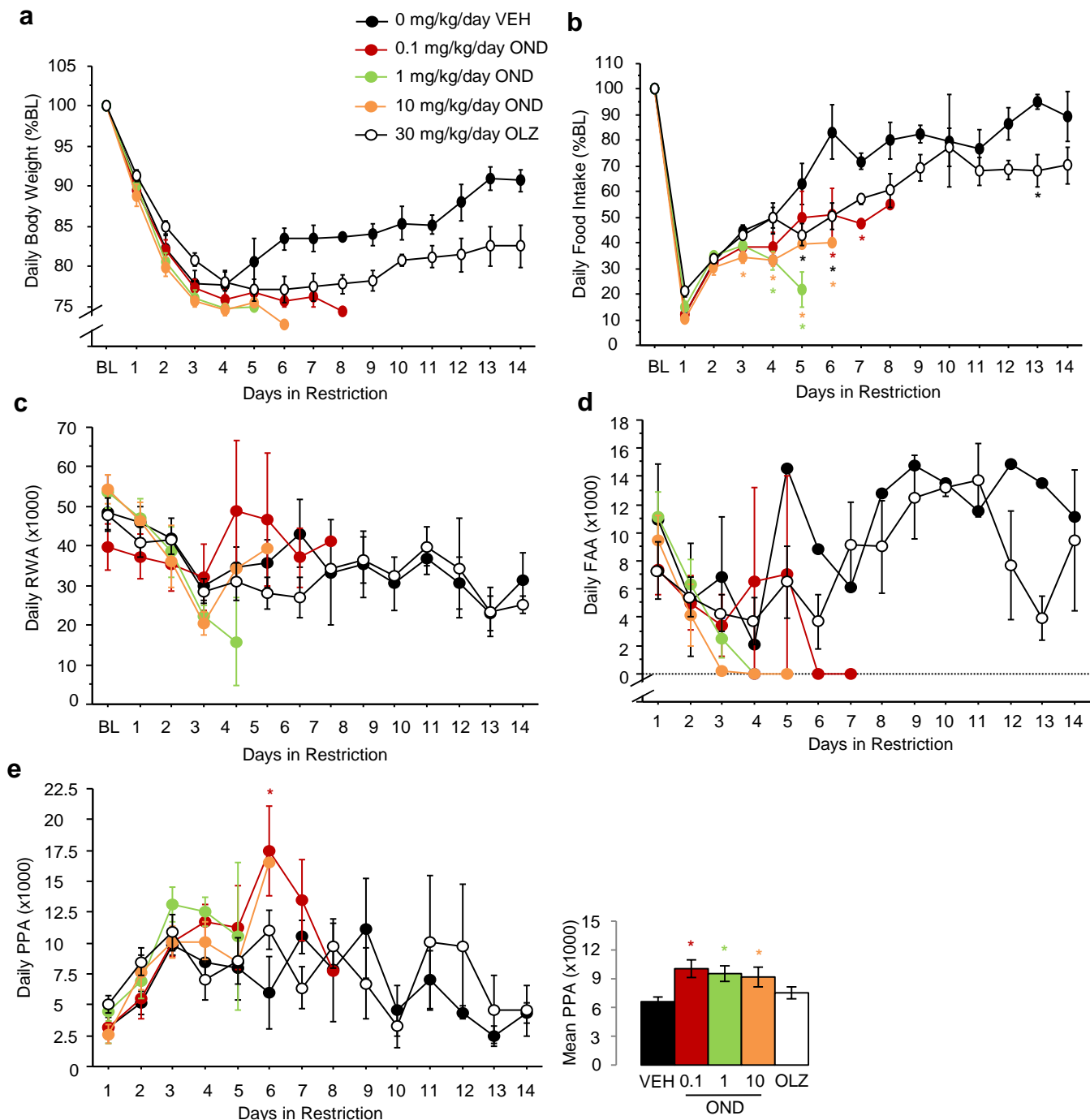

Supplement: Supplementary Figure 2 [file tp2015109x6.pdf]

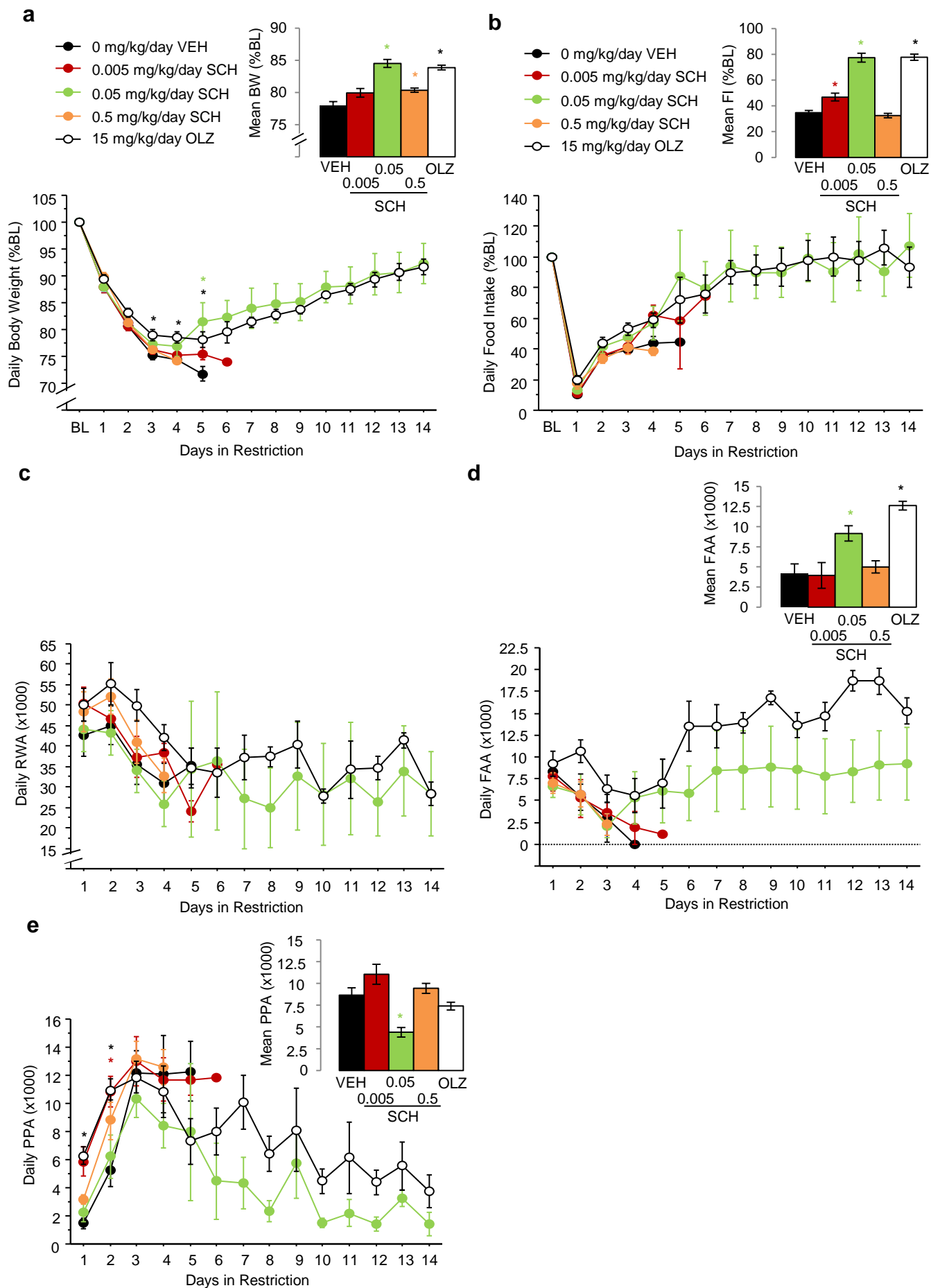

Supplement: Supplementary Figure 3 [file tp2015109x7.pdf]

**a**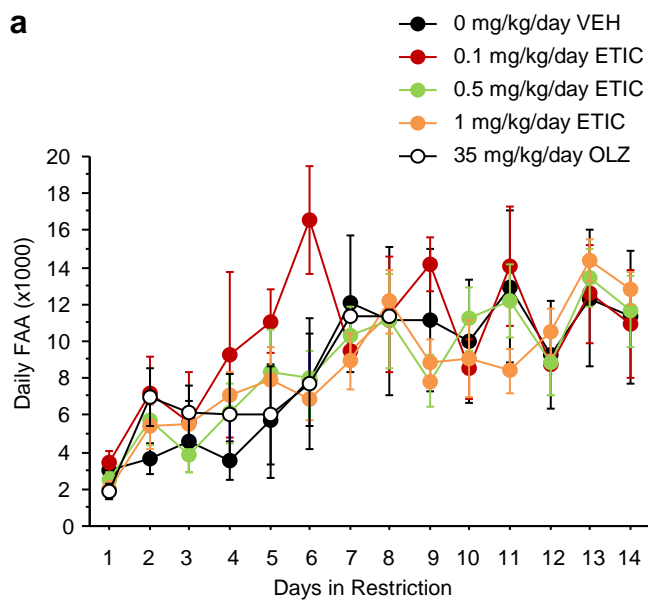**b**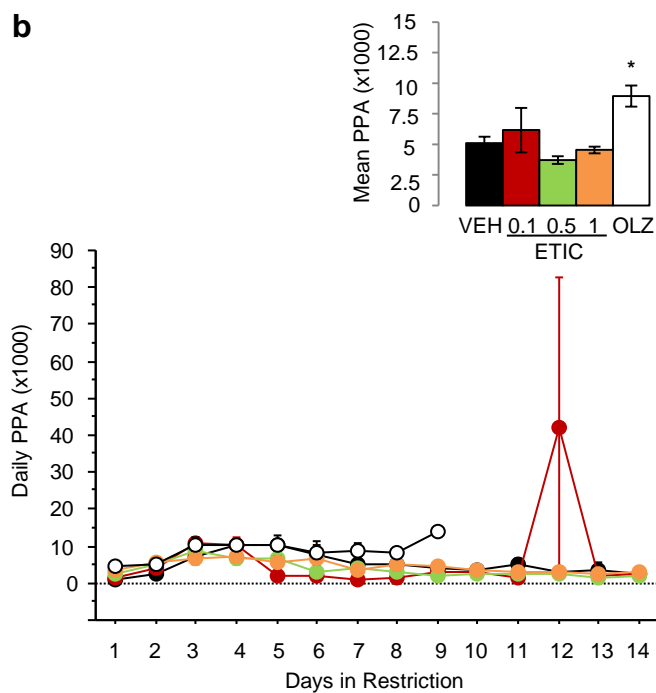

Supplement: Supplementary Figure 4 [file tp2015109x8.pdf]

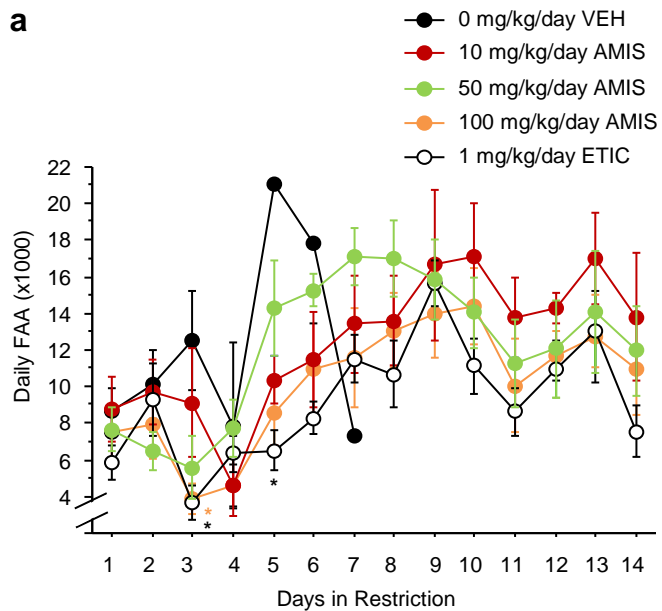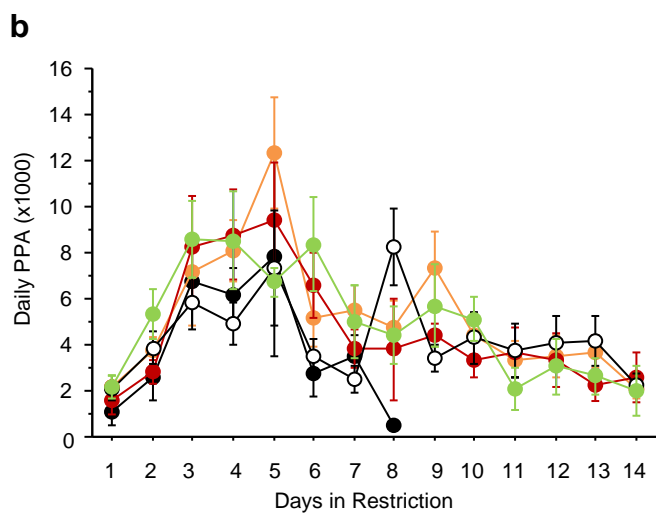

Supplement: Supplementary Figure 5 [file tp2015109x9.pdf]

**a**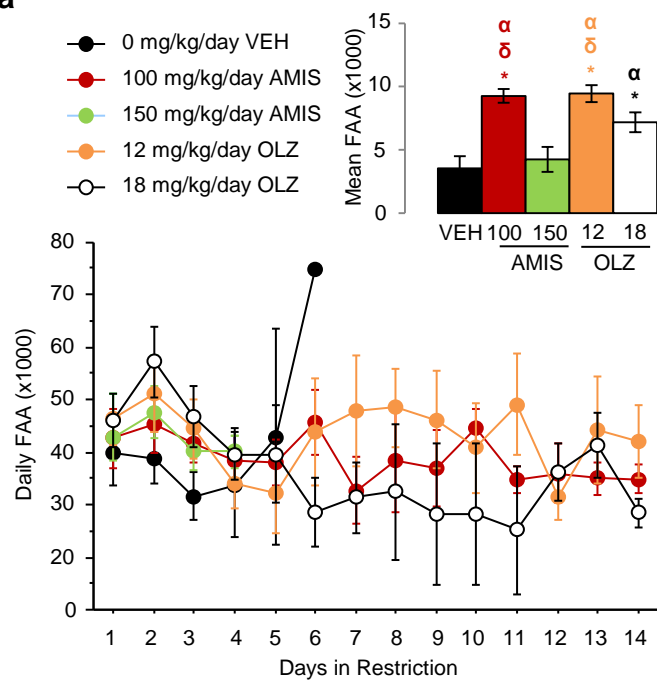**b**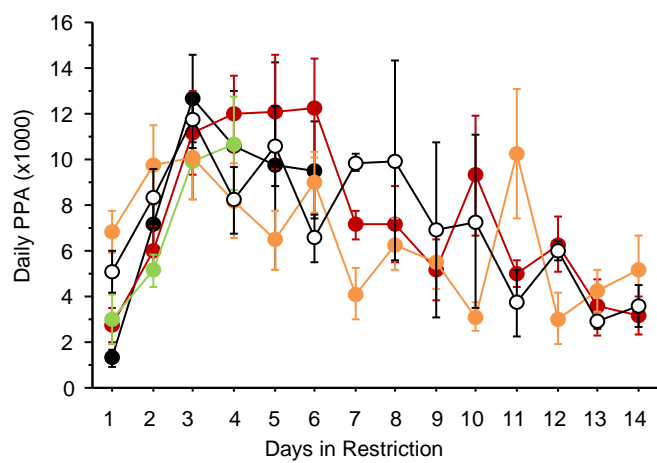

Supplement: Supplementary Figure 6 [file tp2015109x10.pdf]

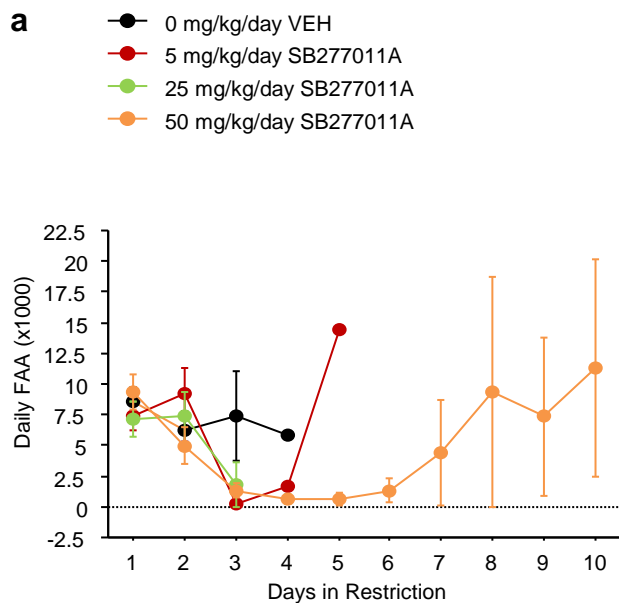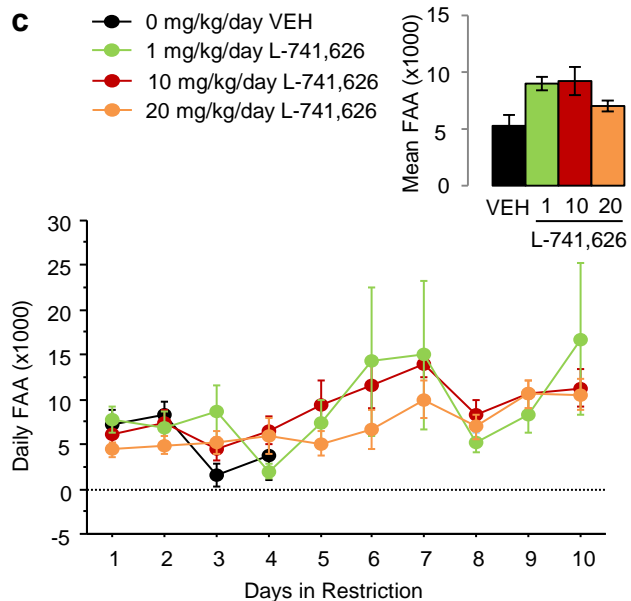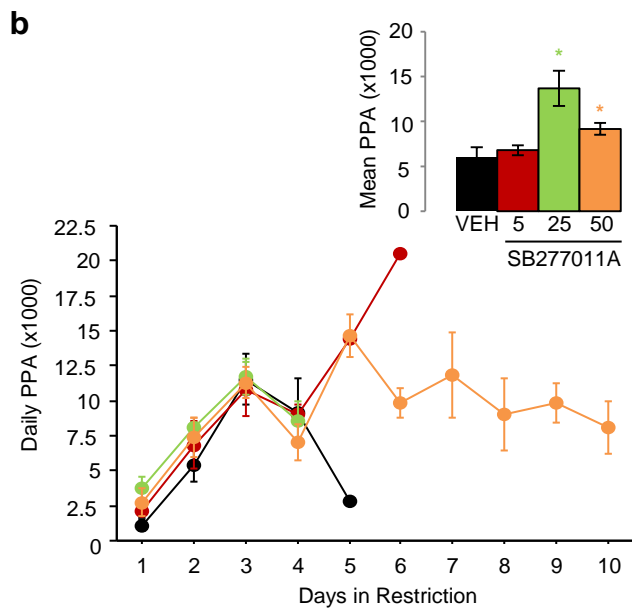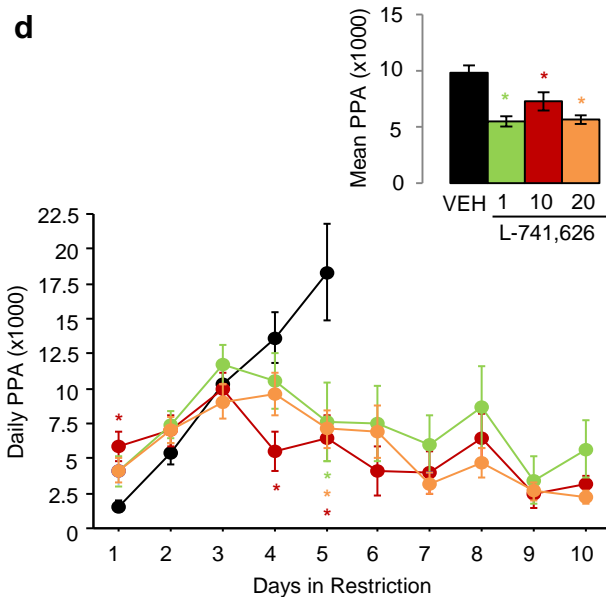

Supplement: Supplementary Figure 7 [file tp2015109x11.pdf]
